# Gut microbiota-derived extracellular vesicles in Alzheimer’s disease—Immunomodulatory mechanisms, biomarkers, and therapeutic opportunities: A review

**DOI:** 10.17305/bb.2025.13213

**Published:** 2025-12-03

**Authors:** Ronghua Yuan, Fei Liu, Jingang Yu

**Affiliations:** 1 School of Nursing, Jiujiang University, Jiujiang, China; 2 Center for Cognitive Science and Transdisciplinary Studies, Jiujiang University, Jiujiang, China; 3 JiuJiang HML Exploratory Healthy-Tech Co., Ltd, Jiujiang University, Jiujiang, China; 4 JiangXi XianKeLai Biotech Co., Ltd, Jiujiang, China; 5 Jiujiang Emergency Medical Center, Jiujiang, China; 6 The Second Affiliated Hospital of Jiujiang University, Jiujiang, China

**Keywords:** Gut microbiota-derived extracellular vesicles, Alzheimer’s disease, neuroinflammation, immune regulation, gut–brain axis, microbiota-immune-neuro axis

## Abstract

Alzheimer’s disease (AD) is a progressive neurodegenerative disorder that poses a growing global health challenge. Beyond traditional hallmarks such as amyloid-β (Aβ) deposition, tau hyperphosphorylation, and neuroinflammation, the gut–brain axis (GBA) has emerged as a significant modulator of AD pathogenesis. Among gut-derived mediators, microbiota-derived extracellular vesicles (mEVs) transport bioactive cargo across epithelial and vascular barriers, thereby linking intestinal dysbiosis to neurodegeneration. This narrative review synthesizes experimental, translational, and early clinical evidence regarding the immunomodulatory roles of gut mEVs in AD. We examine how mEVs may traverse compromised intestinal and blood–brain barriers, activate microglia and astrocytes, and influence Aβ and tau metabolism, thereby integrating peripheral and central immune interactions. Based on this evidence, we propose the “microbiota–EV–immune–neuro axis” as a conceptual framework that connects gut dysbiosis with AD-related neurodegeneration. The review also highlights emerging data on mEV signatures as minimally invasive biomarkers and explores their potential as therapeutic targets or delivery vectors. While current evidence is preliminary and methodologically heterogeneous, mEVs are increasingly recognized as both indicators and potential modulators of AD pathophysiology, emphasizing the need for standardized, longitudinal, and interventional studies.

## Introduction

Alzheimer’s disease (AD) is the most prevalent form of dementia, characterized by progressive cognitive decline and neurodegeneration [1]. Despite significant advancements in neuroscience, the exact mechanisms underlying AD remain inadequately understood. Traditional hypotheses have focused on amyloid-β (Aβ) accumulation, tau hyperphosphorylation, synaptic dysfunction, and chronic neuroinflammation, all of which contribute to neuronal degeneration and cognitive impairment [2]. However, existing therapies targeting these established pathways have demonstrated limited clinical efficacy, indicating that additional systemic factors may influence the onset and progression of the disease.

Recently, the gut–brain axis (GBA) has emerged as a bidirectional network that integrates gut microbiota, immune function, and neural signaling [3]. Alterations in gut microbial composition have been linked to systemic inflammation, metabolic imbalance, and neurodegeneration, suggesting a broader immunometabolic basis for AD [4]. Among the various mediators connecting the gut and brain, microbiota-derived extracellular vesicles (mEVs)—nanosized particles secreted by gut bacteria that contain lipids, proteins, and nucleic acids—have surfaced as a novel and biologically active mode of communication. These vesicles can traverse epithelial and vascular barriers, enter systemic circulation, and potentially influence central immune responses. Their ability to modulate inflammation, oxidative stress, and neuronal homeostasis positions them as significant contributors to the pathophysiology of AD.

Growing experimental and clinical evidence supports a role for mEVs in linking gut dysbiosis and neuroinflammation. Preclinical studies demonstrate that bacterial vesicles can cross the blood–brain barrier (BBB) and activate microglia via toll-like receptor pathways. Early human data suggest that mEV profiles in feces and plasma differ between individuals with AD and cognitively normal controls. Collectively, these findings indicate that mEVs may function as both messengers and mediators within a “microbiota-EV-immune-neuro” network.

The objective of this review is to summarize current evidence regarding the immunomodulatory roles of gut mEVs in the pathogenesis of AD. We will discuss how these vesicles influence immune regulation, amyloid and tau metabolism, and neuroinflammatory cascades, while also considering their diagnostic and therapeutic potential.

## Methods

### Search strategy

This review aims to summarize and evaluate current evidence on the immunomodulatory functions of gut microbiota-derived extracellular vesicles (mEVs) in AD. A comprehensive literature search was conducted on PubMed, Web of Science, Embase, and Scopus for publications up to July 2025. The following keyword combinations were employed: “Alzheimer’s disease” or “dementia,” “gut microbiota” or “intestinal microbiome,” and “extracellular vesicles,” “bacterial outer membrane vesicles,” or “microbiota-derived vesicles.” These terms were further combined with keywords related to “neuroinflammation,” “immune modulation,” or “microglia.”

Reference lists of eligible articles and recent reviews were manually screened to identify additional relevant studies. Only peer-reviewed journal articles were included, and there were no language restrictions at the initial stage.

### Study selection

All retrieved records were imported into reference management software, and duplicates were removed. Titles and abstracts were screened to exclude irrelevant studies, such as those not directly related to AD or those focusing solely on host-derived exosomes. Full-text evaluations were then performed to confirm eligibility.

Studies were included if they examined extracellular vesicles (EVs) produced by gut or commensal microbes and explored their roles in AD or neurodegenerative processes associated with inflammation, amyloid or tau metabolism, BBB function, or neuronal injury. Both experimental and human studies were considered eligible, including *in vivo*, *in vitro*, or *ex vivo* mechanistic studies, as well as observational or interventional clinical research.

Studies were excluded if they (1) did not involve microbial extracellular vesicles; (2) lacked outcomes related to AD; (3) were conference abstracts, commentaries, or editorials without original data; or (4) provided insufficient methodological information to assess study quality. Disagreements during the selection process were resolved through discussion among the authors.

### Data extraction and synthesis

Each included study was systematically reviewed to extract key information, including publication year, study design, model system, microbial source of vesicles, experimental or clinical context, and main outcomes. For animal and cell studies, data on vesicle preparation, dosage, and routes of administration were documented alongside reported effects on immune and neural parameters. In human studies, participant characteristics, analyzed biological samples (plasma, feces, cerebrospinal fluid), and identified vesicle biomarkers were summarized. Due to the heterogeneity among study designs and outcome measures, results were summarized narratively rather than statistically aggregated. The synthesis was organized thematically according to major biological mechanisms discussed in the main text, including vesicle transport across biological barriers, activation of innate immune pathways, modulation of neuroinflammation, and implications for diagnostic or therapeutic development. This approach facilitated comparison across experimental and human findings while highlighting methodological consistencies and limitations.

### Quality assessment

The methodological quality of the included literature was assessed using criteria appropriate to each study type. For *in vitro* and *ex vivo* studies, emphasis was placed on the characterization of vesicle preparations, validation of purity, and functional assays that supported mechanistic conclusions. Human studies were evaluated based on their cohort designs, with a focus on sample selection, control of confounding variables, and measurement reliability.

Studies identified as having significant methodological deficiencies or incomplete reporting were discussed separately in the review but were excluded from the mechanistic synthesis. This approach ensured that the conclusions of the present review were grounded in the most reliable and reproducible evidence currently available.

### Ethics approval

As this work synthesized data from previously published studies, no new ethical approval or patient consent was required.

## Pathological features and immune mechanisms of AD

### Classical pathological processes

AD is neuropathologically characterized by two principal hallmark proteinopathies: the extracellular accumulation of Aβ peptides forming senile plaques and the intracellular aggregation of hyperphosphorylated tau protein into neurofibrillary tangles [5]. Aβ arises from the sequential enzymatic cleavage of amyloid precursor protein (APP), while tau serves as a microtubule-associated protein crucial for maintaining cytoskeletal stability and facilitating axonal transport [6]. Disruptions in the normal turnover or post-translational regulation of these proteins induce conformational changes that confer neurotoxicity, leading to progressive neuronal dysfunction and death [7].

According to the widely accepted amyloid cascade hypothesis, excessive Aβ production or inefficient clearance leads to its accumulation in the extracellular space, resulting in plaque formation that disrupts synaptic signaling and activates neuroinflammatory cascades [8]. Concurrently, tau undergoes abnormal phosphorylation, detaches from microtubules, and assembles into insoluble fibrils [9]. The resultant tangles interfere with intracellular trafficking and contribute to cytoskeletal collapse, synaptic failure, and neuronal degeneration [10].

Although amyloid plaques and neurofibrillary tangles remain diagnostic cornerstones of AD, emerging evidence suggests that early synaptic dysfunction, mitochondrial impairment, and programmed neuronal apoptosis may occur long before overt structural pathology becomes evident [11]. These early molecular disturbances are now recognized as critical events in the initiation and amplification of the neurodegenerative process characteristic of AD [12].

### The central role of neuroinflammation

Neuroinflammation has emerged as a pivotal pathological feature of AD, rather than merely a secondary consequence of neuronal injury [13]. Microglia, the resident immune cells of the central nervous system (CNS), are swiftly activated by Aβ plaques and hyperphosphorylated tau aggregates. Upon activation, these cells release pro-inflammatory mediators, including interleukin-1β (IL-1β), tumor necrosis factor-alpha (TNF-α), and reactive oxygen species [14]. The sustained presence of these mediators contributes to neuronal stress, synaptic dysfunction, and the establishment of a self-perpetuating inflammatory environment. Chronically activated microglia exhibit diminished phagocytic efficiency, impairing Aβ clearance and exacerbating plaque accumulation [15].

Recent studies indicate that the inflammatory activation associated with AD extends beyond the CNS. Peripheral immune signals, including circulating cytokines and pathogen-associated molecular patterns, can modulate central immune tone and microglial reactivity [16]. This interaction is facilitated by the BBB, a selectively permeable interface that typically maintains central immune privilege [17]. Under pathological conditions such as aging, metabolic disorders, or chronic systemic inflammation, BBB permeability may become compromised, allowing peripheral immune components to infiltrate the brain parenchyma. This creates a bidirectional loop in which systemic inflammation enhances microglial activation, which in turn amplifies neuroinflammation within the CNS [18].

The concept of peripheral-to-central inflammatory signaling offers a mechanistic framework for investigating gut-mediated immune influences on the brain. mEVs may exploit these compromised barriers to deliver immunogenic molecules and modulate neuroinflammatory pathways. Understanding the gut–immune–brain communication axis may unveil novel therapeutic strategies targeting both systemic and central drivers of AD pathology.

## Structural and functional characteristics of gut mEVs

Gut mEVs are nanoscale, bilayered particles secreted by both Gram-negative and Gram-positive bacteria [19]. In Gram-negative species, outer membrane vesicles (OMVs) bud outward from the bacterial outer membrane, while Gram-positive bacteria generate vesicles through localized cell wall remodeling and turgor-driven release. These vesicles typically range from 20 to 250 nm in diameter and are enclosed by negatively charged lipid bilayers that incorporate characteristic bacterial surface components, such as lipopolysaccharide (LPS), lipoteichoic acids, and outer membrane protein A (OmpA) [20, 21].

In addition to their structural organization, mEVs carry diverse molecular cargos, including proteins, lipids, nucleic acids, and metabolites, which collectively govern their biological activity. These cargos enable mEVs to participate in microbe-host communication, immune modulation, and metabolic and neuroactive signaling. Of particular relevance to AD, vesicular cargos such as LPS and microbial RNA fragments are increasingly recognized as potent activators of innate immune pathways, linking gut dysbiosis with neuroinflammatory cascades (Table 1).

**Table 1:** Representative cargo categories of gut microbiota-derived mEVs and their biological significance

**Cargo category**	**Representative molecules/source**	**Putative biological functions**
Proteins	OmpA, lysozymes, lipoproteins	Modulate epithelial barrier integrity; participate in microbial recognition
Lipids	LPS, phospholipids, lipopeptides	Trigger host immune alertness; stabilize vesicle structure
Nucleic acids	miRNA-like fragments (e.g., from E. coli, Bacteroides)	Interfere with host gene expression; may influence inflammatory gene programs
	sRNAs, tRNA-derived fragments	Internalized by host cells; regulatory functions under investigation
Metabolites	Short-chain fatty acids (SCFAs: butyrate, propionate), secondary bile acids, indole derivatives	Regulate metabolic tone; modulate immune homeostasis; affect neuroactive signaling pathways

Abbreviations: MEVs: Microbiota-derived extracellular vesicles; OmpA: Outer membrane protein A; LPS: Lipopolysaccharide; miRNA: MicroRNA; sRNAs: Small RNAs; tRNA: Transfer RNA; SCFAs: Short-chain fatty acids.

Upon release, mEVs can interact with host systems through several routes. They are readily internalized by intestinal epithelial cells via endocytosis or membrane fusion, influencing epithelial permeability and mucosal immune tone [22]. Under pathological conditions, including chronic inflammation, metabolic stress, or aging, the intestinal barrier becomes more permeable, allowing vesicles to translocate into systemic circulation. In this context, circulating mEVs act as long-range communication vectors between the gut and peripheral organs, potentially influencing the CNS, thereby mediating the molecular dialogue underlying GBA dysfunction in AD [23].

## Immunomodulatory mechanisms of mEVs in AD

### From gut to brain: How mEVs may reach and disrupt the CNS

In AD, the pathophysiological significance of mEVs extends beyond general immunomodulation to encompass a potential role in linking intestinal dysbiosis with central neuroinflammation and protein homeostasis disruption. Under conditions of gut barrier compromise, such as aging, low-grade inflammation, or epithelial tight-junction dysregulation, mEVs released by commensal or opportunistic gut microbes can gain access to systemic circulation [22]. Once in circulation, mEVs may interact with the vascular endothelium, including the BBB. Increased BBB permeability, observed in both aging and AD, could facilitate the passage of mEVs into the CNS via paracellular leakage or transcytosis [24]. Preclinical data suggest that vesicles derived from gut bacteria can access distal organs, carrying outer-membrane LPS, lipoproteins, nucleic acids, and small RNAs as bioactive cargo [25].

Once in the bloodstream, mEVs may interact with the vascular endothelium of the BBB through multiple pathways. One pathway involves paracellular passage through endothelial tight junctions when the expression of claudin-5 and occludin is downregulated, a phenomenon observed in aging and neurodegenerative states [26]. A second pathway involved in transcytosis is receptor-mediated transcytosis. In this process, surface ligands of mEVs, such as bacterial lipoproteins or LPS, can bind to endothelial Toll-like receptor 4 (TLR4) in conjunction with cluster of differentiation 14 (CD14) and myeloid differentiation factor 2 (MD-2) complexes. This interaction activates MyD88/NF-κB signaling, leading to enhanced vesicle internalization through clathrin-mediated and caveolin-mediated mechanisms, ultimately resulting in the release of mEVs on the abluminal side of the BBB [27]. In certain models, vesicle exposure also appears to enhance endothelial vesicle secretion and local inflammation, thereby amplifying barrier leakiness [28].

Upon entering the brain parenchyma, mEVs engage resident glial and neuronal cells. Microglia detect vesicle-bound LPS or lipoprotein cargo via TLR4 (and potentially TLR2), triggering MyD88-dependent NF-κB activation, production of pro-inflammatory cytokines (IL-1β, TNF-α, IL-6), and reactive oxygen species [29]. This activated microglial phenotype can diminish phagocytic clearance of Aβ and contribute to synaptic pruning and neuronal damage [30]. Astrocytes exposed to mEVs may respond by increasing complement (C3) expression and chemokine release (e.g., CCL2), which recruits peripheral immune cells and exacerbates neuroinflammatory circuits [31].

Furthermore, mEV cargo may directly influence Aβ and tau metabolism. Small RNA and miRNA-like fragments within vesicles are predicted to target mRNAs encoding APP, β-secretase (BACE1), presenilin-1 (PSEN1), and tau-kinases such as glycogen synthase kinase-3β (GSK-3β) and cyclin-dependent kinase 5 (CDK5) [32]. Moreover, vesicle endocytosis by neurons may induce mitochondrial dysfunction, increase intracellular calcium levels, activate stress-kinase cascades (e.g., JNK/p38), and promote tau hyperphosphorylation and subsequent aggregation [33]. These dual mechanisms—immune-mediated reduction of Aβ clearance and direct modulation of protein-processing gene networks—create a plausible mechanistic link between gut microbiota-derived vesicles and hallmark AD pathology.

**Figure 1.**
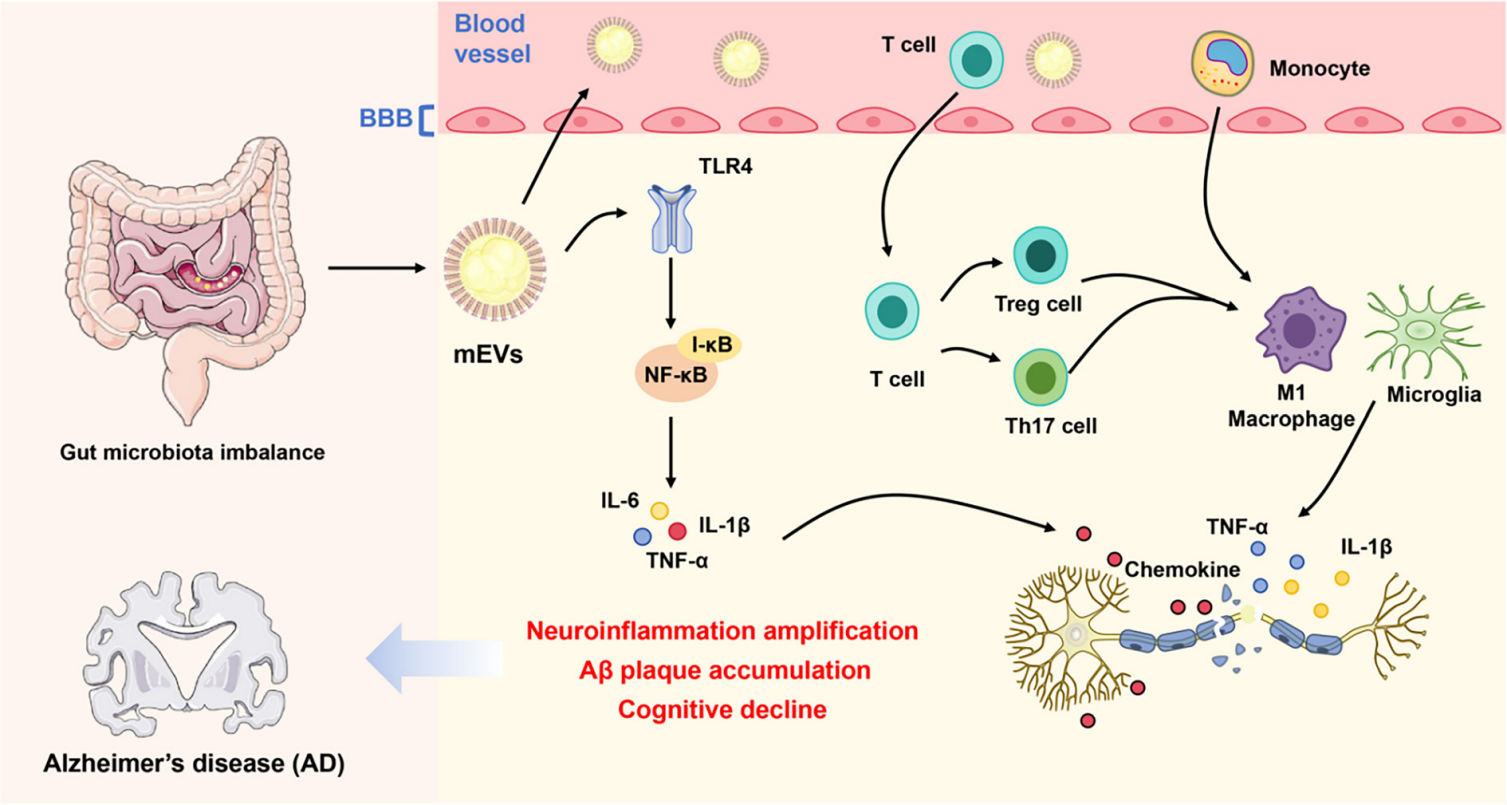
**The immunomodulatory mechanism of gut mEVs in AD.** An imbalance in gut microbiota leads to an increase in circulating mEVs, which traverse the intestinal and blood-brain barriers. This process activates TLR4-NF-κB signaling, resulting in the secretion of pro-inflammatory cytokines, including IL-6, IL-1β, and TNF-α. These inflammatory responses disrupt T-cell homeostasis (Th17/Treg), promote the activation of microglia and M1 macrophages, exacerbate neuroinflammation, and contribute to the accumulation of Aβ plaques and subsequent cognitive decline. Abbreviations: AD: Alzheimer’s disease; mEVs: Microbiota-derived extracellular vesicles; TLR4: Toll-like receptor 4; NF-κB: Nuclear factor kappa-light-chain-enhancer of activated B cells; TNF-α: Tumor necrosis factor alpha; Th17: T-helper 17 cell; Treg: Regulatory T cell; Aβ: Amyloid-beta.

In summary, the available evidence delineates a coherent mechanistic sequence connecting intestinal dysbiosis to central pathology. Disruption of the gut barrier facilitates the release and systemic spread of bacterial vesicles, which can interact with the vascular endothelium and penetrate the BBB. Once within the brain, these vesicles activate microglia and astrocytes, amplify neuroinflammatory signaling, and disrupt the balance of Aβ and tau metabolism through both immune and post-transcriptional pathways (Figure 1). Although many of these findings rely on experimental models and human data remain limited, emerging evidence provides a plausible biological framework linking gut microbiota to neurodegeneration in AD.

### Peripheral-central immune feedback loops

In addition to their direct effects within the CNS, gut-mEVs appear to modulate peripheral immune homeostasis, which may in turn influence neuroinflammatory processes. Dysbiosis of the gut microbiota alters the balance of T-helper 17 (Th17) cells and regulatory T cells (Tregs), with a shift toward Th17-dominant responses linked to increased systemic inflammation and impaired immune regulation [34]. While much of the existing research has focused on microbial metabolites (such as short-chain fatty acids and ATP) rather than vesicles, emerging evidence suggests that mEVs may deliver bacterial antigens, lipoproteins, or small RNAs to antigen-presenting cells in the gut-associated lymphoid tissue. This delivery promotes Th17 differentiation via IL-6/IL-23 signaling and suppresses Treg development through reduced IL-10/TGF-β levels [35]. Once activated, this peripheral pro-inflammatory environment may influence central immune dynamics through multiple pathways.

Systemic inflammatory mediators, including IL-17, IL-1β, TNF-α, and microbial-derived antigens carried within mEVs or released in response to vesicle-stimulated immune activity, can compromise the integrity of the BBB and endothelial signaling [35]. Endothelial cells exposed to IL-17 or bacterial vesicles upregulate adhesion molecules (VCAM-1, ICAM-1) and secrete chemokines (CCL2), facilitating leukocyte trafficking and increasing permeability. Furthermore, mEVs may interact with endothelial TLR4 or receptor for advanced glycation end products (RAGE) receptors, activating NF-κB and the NOD-like receptor family pyrin domain-containing 3 (NLRP3) inflammasome [36], thereby further increasing barrier permeability and enabling the influx of peripheral immune effectors or additional vesicular traffic into the CNS. This mechanism establishes a bidirectional feedback loop: peripheral immune activation influences central glial responses, which in turn amplify systemic inflammation through cytokine spillover.

Based on this understanding, we propose the “microbiota-mEV-immune-neuro axis” as a conceptual framework for the progression of AD: gut microbial vesicles prime peripheral immune responses (altering the Th17/Treg balance), systemic inflammation and vesicle trafficking compromise barrier and vascular signaling, and the resulting glial activation and neuroinflammation accelerate proteinopathies (such as Aβ and tau) and neurodegeneration. Although direct evidence of mEV-mediated Th17/Treg modulation in AD remains limited, the convergence of vesicle biology, immune-axis dysregulation, and neurodegenerative pathology supports this integrative model.

## Experimental evidence linking mEVs to AD

### Evidence from animal models

Animal studies have provided important preliminary insights into how mEVs may influence cognitive function and neuropathological changes under controlled experimental conditions. In one investigation, systemic administration of OMVs derived from *Escherichia coli* in mice resulted in measurable cognitive impairment, accompanied by increased hippocampal amyloid deposition and enhanced microglial activation [37]. These findings suggest that bacterial EVs (bEVs) can cross physiological barriers and engage neuroinflammatory mechanisms.

Similarly, mEVs derived from dysbiotic microbiota have been shown to induce pro-inflammatory phenotypes in glial cells when applied to murine brain slices or following intracerebroventricular injection. In germ-free mouse models, colonization with bacterial strains that secrete vesicles enriched in LPS or miRNA-like molecules has been associated with elevated expression of cytokines such as IL-6 and TNF-α in both peripheral and central tissues [38]. Collectively, these results suggest that mEVs may function as mediators linking microbial imbalance to immune dysregulation, neuroinflammation, and pathological protein aggregation characteristic of AD.

Despite these advances, several limitations must be acknowledged when interpreting data from animal models. Many studies employ supraphysiological doses of vesicles that likely exceed natural exposure levels, thereby limiting biological relevance. Additionally, considerable variability in vesicle purification methods and source materials introduces uncertainty regarding purity and reproducibility. Behavioral assays utilized to assess cognitive outcomes in rodents also possess inherent constraints and may not adequately capture the multifactorial and progressive nature of human AD. Therefore, current evidence from animal models should be viewed as indicative rather than conclusive, providing a foundation for more physiologically relevant investigations in the future.

### Preliminary findings in human studies

In humans, evidence implicating mEVs in AD remains exploratory, largely due to methodological challenges in isolating and accurately characterizing bacterial vesicles from complex clinical samples. Nevertheless, several cross-sectional investigations have reported distinct alterations in fecal EV profiles between individuals with AD and cognitively healthy controls, including changes in lipid composition, inflammatory activity, and RNA cargo content [39–42]. Moreover, specific microbial miRNA fragments detected in plasma-derived EVs have been shown to correlate with systemic inflammatory markers and cognitive performance scores, although these associations do not yet establish causality [22]. A small-scale clinical study further observed that AD patients exhibited elevated circulating EVs expressing bacterial markers such as LPS-binding protein (LBP), a finding that may reflect increased gut permeability or enhanced microbial translocation across mucosal barriers [43]. Despite these promising observations, several factors continue to limit interpretation. Human studies generally involve small cohorts and cross-sectional designs, which restrict statistical power and preclude causal inference. Furthermore, confounding variables, including age, diet, medication exposure, and comorbid conditions, are often insufficiently controlled, potentially obscuring true biological associations. Methodological inconsistency in vesicle isolation and profiling also remains a significant concern; techniques such as ultracentrifugation, immunoaffinity capture, and polymer-based precipitation yield variable results regarding yield and purity, complicating reproducibility and inter-study comparisons. These limitations are summarized in Table 2, which outlines representative findings from animal and human investigations alongside their respective methodological constraints. In conclusion, current human data provide only preliminary yet biologically meaningful indications that mEVs may participate in AD pathophysiology. Although definitive mechanistic evidence is lacking, the convergence of animal and human observations supports the plausibility of mEVs acting as both mediators and biomarkers of neurodegenerative processes, underscoring the importance of longitudinal and interventional studies to clarify their causal relevance in disease progression.

**Table 2:** Summary of experimental evidence linking gut microbiota-derived mEVs to Alzheimer’s disease

**Study type**	**Model/Sample**	**Key observations**	**Limitations**	**References**
Animal (*in vivo*)	Mice injected with *E. coli*-OMVs	Cognitive deficits; ↑ hippocampal Aβ; microglial activation	High-dose exposure; species-specific effects	Wei et al., 2025; Mol Neurobiol.
Animal (*ex vivo*/*in vitro*)	Murine brain slices; glial cultures	Pro-inflammatory activation induced by dysbiotic mEVs	May not replicate *in vivo* physiology	Choi et al., 2022; J Alzheimer’s Dis. Yang et al., 2024; BMC Microbiol.
Human (fecal EVs)	Cross-sectional cohorts (AD vs controls)	Altered lipid and RNA cargo; ↑ inflammatory potential	Small sample sizes; cross-sectional; confounders	Lin et al., 2025; Front Neurosci.
Human (plasma EVs)	AD patients vs controls	↑ LPS-binding protein–positive EVs; microbial RNAs linked to cognition	Correlative only; EV origin hard to verify	Li et al., 2024; Nature aging

↑: Increased. Abbreviations: AD: Alzheimer’s disease; Aβ: Amyloid-β; OMVs: Outer membrane vesicles; mEVs: Microbiota-derived extracellular vesicles; EVs: Extracellular vesicles; LPS: Lipopolysaccharide.

## Diagnostic and therapeutic potential of mEVs in AD

As research on AD increasingly embraces systems-level approaches, mEVs have emerged as pivotal mediators of communication between the gut and the brain. Their capacity for systemic circulation, interaction with immune and endothelial pathways, and ease of detection in accessible biofluids positions them as promising candidates for early diagnosis, therapeutic modulation, and engineered delivery in AD.

### Biomarker potential

mEVs exhibit significant potential as non-invasive biomarkers. Their lipid bilayer encapsulation safeguards diverse molecular cargo, including microbial RNAs, lipids, and surface proteins, thereby ensuring stability under physiological conditions [44]. Moreover, mEVs can be retrieved from plasma, feces, and cerebrospinal fluid [45]. In practice, plasma and fecal sampling are the most scalable methods for serial testing. Methodological advancements have enhanced analytical specificity; for instance, selective recovery of LPS-positive bEVs from feces and plasma has been demonstrated through various characterization techniques, including nanoparticle tracking analysis, immunogold transmission electron microscopy (TEM), flow cytometry, super-resolution microscopy, and 16S profiling. These methods provide a pathway to enrich microbiota-derived signals from heterogeneous vesicle populations [46]. Emerging clinical and translational evidence supports the diagnostic utility of EV profiling in AD. A 2024 study examining plasma EV-derived mRNA in 82 individuals (including healthy controls, those with mild cognitive impairment, and AD patients) reported a diagnostic model with an area under the receiver operating characteristic (ROC) curve exceeding 0.98 [47]. Although this analysis focused on total plasma EVs rather than specifically on microbiota-derived vesicles, it offers an important methodological framework that can be adapted for assays targeting microbiota-derived EVs. Recent workflow reviews further affirm that EVs serve as accessible biomarker vehicles in neurodegenerative diseases and outline critical steps—from pre-analytical sample handling to library preparation and data integration—that are directly applicable to mEV investigations [48, 49]. Recent mechanistic studies have solidified the connection between bacterial vesicle biology and AD-related pathology. *In vivo* research has demonstrated that gut-derived bEVs enriched in LPS can traverse the BBB, activate microglia, and initiate complement and mechanosensory pathways, contributing to synaptic injury and neuronal loss [50]. These findings provide a biological basis for detecting disease-associated mEV signatures in peripheral fluids such as blood or stool. Concurrent advances in biosensing and microfluidic technologies indicate that EV-based point-of-care diagnostic systems are now technically feasible. Recent efforts employing surface plasmon resonance and nanoelectronic biosensors have achieved sensitive detection of AD-related EV cargo, suggesting the potential for similar methodologies to be adapted for microbiota-derived vesicle targets once capture ligands and antibody panels are standardized [51]. Comprehensive analyses of microbiota-derived vesicles continue to enhance our understanding of cargo composition and host interaction patterns, illuminating the diversity of outer-membrane ligands, lipids, and small RNAs that mediate immune and neuroinflammatory signaling [25]. These studies provide essential guidance for selecting candidate molecular markers in future mEV-based assays. However, a significant practical challenge in translating these findings is the accurate differentiation of bacterial vesicles from the more prevalent host-derived EVs in human biofluids. Due to overlapping size and density distributions, effective separation necessitates targeted enrichment for bacterial markers such as LPS or outer membrane proteins, along with proteomic or genomic validation [52]. Furthermore, the absence of standardized omics workflows—from sample collection and RNA extraction to normalization and data integration—introduces substantial variability across laboratories [53]. Consequently, the establishment of standardized reference materials and cross-platform validation schemes is essential to ensure the reproducibility and comparability of future biomarker data. Despite recent advancements in understanding microbiota-derived vesicles, several practical challenges persist that hinder their clinical application. Isolation methods remain inconsistent, with techniques such as ultracentrifugation, size-exclusion chromatography, and immunoaffinity capture yielding variable recovery and purity across different laboratories. Additionally, the molecular composition of mEVs is highly context-dependent, influenced by dietary factors, medications, microbial diversity, and circadian rhythms, complicating the establishment of stable disease signatures. Diagnostic specificity is another challenge, as certain RNA and lipid profiles overlap with those observed in other chronic inflammatory or metabolic disorders. Addressing these challenges will require the development of harmonized pre-analytical workflows and enrichment strategies capable of distinguishing bacterial vesicles from host-derived counterparts, such as LPS-directed immunocapture. Moreover, the integration of multi-omics datasets will be crucial for enhancing analytical reproducibility. Once standardized, mEV-based biomarkers could transition from exploratory findings to clinically validated diagnostic tools, complementing the broader EV platforms currently being developed for neurodegenerative disease research.

### Therapeutic targeting strategies

mEVs impact AD pathology through their secretion, systemic transport, and interaction with host immune and neural pathways, each representing a potential therapeutic target. From a microbiological perspective, modulating gut composition to reduce the release of pro-inflammatory vesicles has demonstrated promising results. Certain bacterial strains, particularly Bacteroides fragilis and Escherichia coli, are known to release LPS-enriched vesicles that exacerbate systemic inflammation and microglial activation [28]. A recent study indicated that gut-derived bacterial vesicles carrying LPS could traverse the BBB, activate microglia, and engage complement signaling in AD models [50]. Therefore, strategies that modify gut microbial communities, such as enhancing probiotic strains or increasing dietary fiber to shift vesicle content toward anti-inflammatory profiles, are plausible. Another approach involves limiting the systemic dissemination of mEVs by fortifying the intestinal barrier or BBB. A study utilizing a microfluidic platform revealed that microbial-derived exosomes and metabolites influence neuronal growth and synaptic plasticity in GBA chip systems, suggesting that barrier integrity is crucial for vesicle entry into the CNS [54]. Nutritional or hormonal interventions that strengthen epithelial or endothelial junctions may help to reduce vesicle load in circulation, although direct evidence in humans remains insufficient. At the host level, disrupting vesicle–host receptor interactions presents another therapeutic avenue. Inhibiting pattern recognition receptors, such as TLR4 and nucleotide-binding oligomerization domain-containing protein 2 (NOD2), which detect components of bacterial vesicles, may reduce downstream immune activation [55]. For instance, a recent investigation into probiotic-derived EVs illustrates how vesicle cargo modulates immune signaling and how engineered vesicles may circumvent inflammatory triggers [56]. The concept of “functional decoupling,” where beneficial commensal microbial signals persist while pro-inflammatory vesicle-driven pathways are mitigated, is compelling yet technically challenging.

Despite these promising avenues, most strategies remain at the preclinical stage. A significant limitation is the scarcity of clinical trials specifically targeting mEV pathways or vesicle-based therapies in AD. Additional obstacles include an incomplete understanding of which vesicle subtypes are pathogenic, variability in vesicle isolation techniques, and host-specific response discrepancies. Future advancements will rely on the integration of microbiome taxonomy, detailed vesicle cargo profiling, and host immune phenotyping into cohesive experimental and clinical frameworks. This integrative approach may ultimately facilitate the rational design of vesicle-targeted or vesicle-based therapies for AD.

### Engineered vesicles and probiotic-derived applications

In addition to efforts aimed at modulating endogenous mEVs, researchers are investigating two complementary therapeutic avenues: engineered vesicles and probiotic-derived vesicles. Engineered or synthetic EVs are being developed as precision carriers for small RNAs, anti-inflammatory compounds, or neuroprotective peptides targeted to specific tissues, such as the intestinal mucosa or CNS [57]. Their lipid bilayer composition provides stability and controlled release, while surface engineering enhances tissue tropism and receptor-specific targeting. Liang et al. [58] demonstrated that engineered exosomes, modified with neuron-targeting ligands and loaded with siRNAs against tau, effectively reduced hyperphosphorylated tau and neuroinflammatory cytokines in mouse models of AD. Similarly, synthetic EV-mimetic nanoparticles encapsulating AMP-activated protein kinase (AMPK) activators have shown potential for restoring mitochondrial metabolism and reducing β-amyloid aggregation in preclinical systems [59]. These findings underscore the viability of using EV scaffolds for targeted, biocompatible neurotherapies. Concurrently, research has focused on vesicles naturally secreted by beneficial probiotic strains. Vesicles derived from Lactobacillus rhamnosus and Bifidobacterium longum attenuate the release of TNF-α, IL-1β, and IL-6, while upregulating IL-10, thereby dampening microglial activation and promoting intestinal-brain homeostasis [60]. Their favorable safety profile and compatibility with microbiome modulation strategies render probiotic-derived vesicles attractive for managing inflammation associated with AD [61]. Zhang et al. [56] further highlighted their postbiotic potential, illustrating how bacterial vesicle lipids and RNA cargo can recalibrate host immune responses without systemic toxicity. A novel concept, *in vivo* vesicle induction, proposes stimulating the host microbiota to produce beneficial mEVs through targeted dietary interventions or microbial consortia. For example, fermentable fibers and specific prebiotics can enhance the release of anti-inflammatory vesicles enriched in short-chain fatty acid pathways, indirectly alleviating neuroinflammation. Verbunt and Stassen [62] described this approach as a controllable “endogenous vesicle bioreactor” strategy, which may circumvent production complexities and minimize immune rejection. Collectively, these strategies, from engineered EV carriers to probiotic and diet-induced vesicles, illustrate the expanding translational landscape of mEVs in AD. They provide integrated opportunities for biomarker development, therapeutic modulation, and precision vesicle-based delivery. A structured overview of representative strategies, experimental evidence, and their comparative advantages and limitations is summarized in Table 3.

**Table 3:** Diagnostic and therapeutic potential of gut microbiota-derived mEVs in Alzheimer’s disease: Current status, advantages, and limitations

**Application area**	**Strategy/Approach**	**Representative evidence**	**Advantages**	**Limitations/challenges**	**Maturity (TRL/Stage)**	**References**
Diagnostics	Plasma/fecal/CSF mEV profiling (miRNAs, lipids, proteins)	Altered mEV profiles in AD vs controls; microbial RNAs linked to cognitive decline	Non-invasive; stable cargo; accessible in multiple biofluids	Heterogeneous isolation methods; small sample sizes; confounders (age, diet, comorbidities); low specificity	TRL 3–4 (exploratory clinical association study)	Lin et al., 2025; Front Neurosci.
Therapeutics: Microbial targeting	Modulate gut microbiota composition (reduce pro-inflammatory strains)	Animal studies showing reduced LPS-rich mEVs improve inflammation	Shifts vesicle profile; leverages microbiome modulation	Strain-specific effects; lack of causal validation	TRL 3–4 (preclinical, *in vivo* proof-of-concept)	Li et al., 2024; Nature Aging
Therapeutics: Barrier function	Enhance intestinal barrier and BBB integrity (prebiotics, nutrition, hormonal modulation)	Animal models show improved barrier reduces systemic inflammation	Indirectly limits mEV trafficking; feasible in lifestyle interventions	Effects on EV trafficking not yet directly proven; variable in humans	TRL 2–3 (animal studies)	Wang et al., 2025; Front Pharmaco.
Therapeutics: Host receptor blocking	Inhibit TLR4/NOD2 signaling (“functional decoupling”)	*In vitro* evidence of dampened inflammatory response to mEVs	Selective targeting of inflammatory pathways	Technical complexity; systemic immune side effects possible	TRL 4–5 (preclinical validation)	Chen et al., 2024; Cell Rep Med.
Engineered vesicles	Synthetic/engineered EVs delivering siRNAs, peptides	Preclinical models (Parkinson’s, MS) show therapeutic benefit	High specificity; customizable cargo	Early-stage; safety and immunogenicity concerns	TRL 2–3 (concept/*in vitro*)	Zhao et al., 2024; Adv Sci.
Probiotic-derived vesicles	EVs from *Lactobacillus*, *Bifidobacterium*	*In vivo* suppression of pro-inflammatory cytokines; cognitive benefit in AD-like models	Biocompatible; safe history of probiotic use	Limited human validation; production scale-up issues	TRL 3–4 (preclinical animal validation)	Verbunt et al., 2025; Microbiome Res Rep.
*In vivo* induction	Stimulate host-compatible strains to produce beneficial EVs via diet/microbial interventions	Conceptual; no direct AD-specific validation yet	Potentially safer, self-sustaining therapeutic effect	Highly speculative; mechanism not defined	TRL 1–2 (conceptual/hypothesis-generating)	Zhang et al., 2025; J Nanobiotechnology

Abbreviations: AD: Alzheimer’s disease; BBB: Blood–brain barrier; CSF: Cerebrospinal fluid; EVs: Extracellular vesicles; mEVs: Microbiota-derived extracellular vesicles; LPS: Lipopolysaccharide; miRNAs: MicroRNAs; siRNAs: Small interfering RNAs; MS: Multiple sclerosis; TLR4: Toll-like receptor 4; NOD2: Nucleotide-binding oligomerization domain-containing protein 2; TRL: Technology readiness level.

## 7a Limitations and future directions

While the translational potential of gut microbiota-derived mEVs in AD is increasingly acknowledged, significant limitations persist. Addressing these challenges is essential for advancing the field from preclinical promise to clinical applicability.

### Current limitations

Most mechanistic insights regarding mEVs in AD are derived from cellular and animal studies. Although these models offer valuable insights into potential signaling pathways, they cannot replicate the full physiological and environmental complexity of the human GBA. Additionally, the composition of mEV cargo is highly variable, influenced by microbial species, host immune status, diet, and other external factors, complicating the establishment of consistent disease-specific patterns. Evidence from human studies remains preliminary and largely correlative. Existing investigations typically involve small cohorts and cross-sectional designs, with limited adjustment for confounding variables such as age, medication, and comorbidities. Consequently, it remains unclear whether the observed changes in mEV profiles are drivers, consequences, or mere epiphenomena of Alzheimer’s pathology. Technical and methodological challenges further impede progress. Reliable differentiation between bacterial and host-derived vesicles remains challenging, and isolation and characterization procedures vary substantially among laboratories. These inconsistencies compromise reproducibility and hinder meaningful comparisons across studies. Moreover, many preclinical experiments utilize supraphysiological vesicle doses or lack standardized reporting of experimental controls, complicating interpretation and translation to human relevance. Lastly, translational and regulatory barriers continue to exist. Current frameworks governing the therapeutic applications of microbial vesicles are underdeveloped, and standards for good manufacturing practices (GMPs) concerning large-scale, stable, and safe production have yet to be established. Ethical considerations surrounding microbiome manipulation and engineered vesicle therapies also necessitate careful evaluation. Collectively, these limitations suggest that while mEVs represent a promising connection between the gut microbiome and neurodegeneration, their causal role in AD remains uncertain. Future research should prioritize improving methodological consistency, expanding well-controlled human cohorts, and establishing standardized analytical pipelines before responsibly pursuing therapeutic translation.

### Future research directions

To enhance the understanding of mEVs in AD, several research priorities should be addressed at technical, mechanistic, and translational levels. In the short term, it is crucial to improve technical consistency and methodological rigor. Standardizing procedures for vesicle isolation and characterization, as well as integrating multi-omics approaches—such as metagenomics, transcriptomics, proteomics, and lipidomics—will enhance data comparability and biological interpretation. The application of advanced *in vitro* organ-on-chip models may complement animal studies by providing controlled environments for investigating vesicle transport and host-cell interactions. In the medium term, strengthening human-based evidence should be a key objective. Well-designed longitudinal cohort studies focusing on at-risk populations, such as individuals with mild cognitive impairment, are necessary to determine whether mEV signatures can predict cognitive decline or disease progression. Mechanistic studies that incorporate host genetic backgrounds, including APOE4 and TREM2 variants, will clarify how genetic susceptibility modifies vesicle-mediated signaling. Concurrently, research into vesicle engineering through microbial strain modification or synthetic vesicle platforms should continue, aiming to develop more precise tools for mechanistic exploration and potential therapeutic applications. Recent advances in analytical technology are elucidating the role of mEVs in AD. The use of spatial transcriptomics is beginning to reveal region-specific inflammatory and metabolic changes in the Alzheimer’s brain, potentially linked to vesicle-mediated signaling [63]. Studies employing single-vesicle multi-omics have demonstrated that individual vesicles in human plasma and cerebrospinal fluid carry distinct RNA and protein signatures associated with disease stage, supporting their potential as precise biomarkers [64]. Therefore, integrating these spatial and single-particle datasets with microbiome-derived information may enable mapping of gut-to-brain communication at the cellular level. Furthermore, the concept of strain-specific vesicle profiling, where vesicle composition is matched to an individual’s gut microbial strains and genetic background, is emerging as a foundation for personalized risk assessment and therapeutic design. Establishing standardized pipelines that combine these methods across human cohorts will be a crucial next step toward reproducible, individualized mEV-based diagnostics and treatment strategies. In the long term, clinical translation will hinge on establishing regulatory standards, scalable production systems, and comprehensive safety evaluations. Developing GMP-compliant manufacturing protocols, reproducible efficacy testing, and thorough immunogenicity assessments will be essential for advancing vesicle-based strategies toward clinical testing. Incorporating bioethical oversight into future trial designs will ensure that emerging interventions are evaluated not only for effectiveness but also for safety and ethical acceptability. Collectively, these steps could transform current experimental insights on mEVs from exploratory findings into reliable, clinically relevant strategies for AD prevention and management.

## Conclusion

Gut mEVs are emerging as key mediators linking the GBA to AD. By traversing biological barriers and carrying immune-active cargo, they provide a mechanistic link between gut dysbiosis, immune modulation, and CNS dysfunction. Current evidence, although still limited, supports two primary research priorities. The first is to elucidate the immunomodulatory role of mEVs in AD, particularly their involvement in peripheral-central immune communication. The second is to transition from mechanistic studies toward translational applications, including their use as biomarkers and potential therapeutic tools. Thus, mEVs offer a novel perspective on the systemic nature of AD and may function as both indicators and modulators of disease processes. Continued efforts to integrate basic research with clinical studies will be essential to realize their potential in diagnosis and treatment.

## Data Availability

The data used to support the findings of this study are available from the corresponding author upon request.
